# Polymorphisms of the Flavin containing monooxygenase 3 (*FMO3*) gene do not predispose to essential hypertension in Caucasians

**DOI:** 10.1186/1471-2350-6-41

**Published:** 2005-12-02

**Authors:** Ciara Dolan, Denis C Shields, Alice Stanton, Eoin O'Brien, Deborah M Lambert, John K O'Brien, Eileen P Treacy

**Affiliations:** 1 Department of Clinical Pharmacology, Royal College of Surgeons in Ireland, 123 St Stephens Green, Dublin 2, Ireland; 2 Conway Institute, University College Dublin, Dublin 4, Ireland; 3 ADAPT Centre, Beaumont Hospital and Department of Clinical Pharmacology, Royal College of Surgeons in Ireland, Dublin 2, Ireland; 4 Department of Genetics, Children's University Hospital, Temple St, Dublin 1, Ireland; 5 National Centre for Inherited Metabolic Disorders, Children's University Hospital, Temple St., Dublin 1, Ireland

## Abstract

**Background:**

The recessive disorder trimethylaminuria is caused by defects in the *FMO3 *gene, and may be associated with hypertension. We investigated whether common polymorphisms of the *FMO3 *gene confer an increased risk for elevated blood pressure and/or essential hypertension.

**Methods:**

*FMO3 *genotypes (E158K, V257M, E308G) were determined in 387 healthy subjects with ambulatory systolic and diastolic blood pressure measurements, and in a cardiovascular disease population of 1649 individuals, 691(41.9%) of whom had a history of hypertension requiring drug treatment. Haplotypes were determined and their distribution noted.

**Results:**

There was no statistically significant association found between any of the 4 common haplotypes and daytime systolic blood pressure in the healthy population (p = 0.65). Neither was a statistically significant association found between the 4 common haplotypes and hypertension status among the cardiovascular disease patients (p = 0.80).

**Conclusion:**

These results suggest that the variants in the *FMO3 *gene do not predispose to essential hypertension in this population.

## Background

Essential hypertension is the most common reason for adult visits to office-based physicians and for the use of medication [1-3]. It is estimated that 24% of the US adult population (70% in persons over 70) and at least 50% of the Irish adult population aged 50 or over are hypertensive[4,5]. Ethnic differences exist in the prevalence of hypertension between populations, for example between Africans and Northern Europeans [6-8]. Mendelian forms of hypertension resulting from single gene defects are rare [9-11]. The major contribution to the etiology of this disorder is proposed to result from the combined effects of genes that modify the response of blood pressure to environmental stresses such as diet and 'environmental susceptibility genes' [12-14]. Essential hypertension does not follow a clear pattern of inheritance but exhibits familial aggregation of cases. This multifactorial trait increases the affected individuals' risk of myocardial infarction, stroke and end-stage renal disease, and is one of the leading causes of morbidity and mortality in adults[8,15]. Population-wide application of preventative measures and candidate gene analysis to predict modifiable risks in addition to treatment are thus very worthwhile [16-19].

Trimethylaminuria (TMAuria) is an inborn error of metabolism (MIM # 275700) resulting from diminished oxidation of the tertiary amine trimethylamine to trimethylamine N-oxide resulting in a severe body odour in affected individuals. The microsomal NADPH-dependent flavin-containing monooxygenases (FMOs) (E.C. 1.14.12.8) are a family of chemical and drug metabolising enzymes that catalyze the phase 1 oxygenation of a wide variety of nucleophilic heteroatom containing compounds including catecholamines[20,21]. *FMO3*, a phase 1 drug metabolising gene, is the main liver dependent human isoform[22]. We and others have shown that recessivity for mutations of *FMO3 *cause TMAuria [20-24].

We have previously noted that a number of patients with TMAuria have idiopathic hypertension [21,22]. In addition, Cashman et al [25] have reported a proposed link in African-American males between hypertension and increased excretion of trimethylamine. We have identified increased excretion of catecholamines in a proband with TMAuria who is homozygous for a deletion of the *FMO3 *gene [20]suggesting a possible association between abnormal catecholamine metabolism and variants of the *FMO3 *gene. Endogenous substrates for FMO3 include tyramine and phenylethylamine [26,27] and tyramine has a known pressor effect. As high levels of circulating catecholamines contribute to hypertension, it is proposed that polymorphisms of *FMO3 *gene could contribute to impaired catecholamine metabolism and hypertension.

We have previously described the population frequencies of a number of common polymorphisms of the *FMO3 *gene (E158K, V257M, E308G) in a North American population[25,28]. Expression studies indicate that the polymorphisms E158K and V257M exhibit decreased tyramine oxidation *in vitro*[25]. We and others have noted that the E308G polymorphism shows a substantial decrease in *FMO3 *activity in the presence of the E158K polymorphism causing mild TMAuria[22,29]. While these common polymorphisms exhibit some difference in expression of the functional FMO3 enzyme, this effect is minimal in comparison to that of the null mutations.

We hypothesised that common *FMO3 *polymorphisms might predispose to essential hypertension. While null alleles in the *FMO3 *gene may be too rare to cause any appreciable effect on the population burden of hypertension, prevalent polymorphisms with documented *in vitro *evidence of variation in catecholamine metabolism may however potentially associate with and predispose to this condition.

Herein we report our investigations into a possible association of haplotypes of three polymorphic variants of the *FMO3 *gene with the phenotype of blood pressure in an occupational Irish adult population and with presence of essential hypertension in an Irish cardiovascular disease (CVD) population.

## Methods

### Study populations

There were 2 population groups in this study. The first population was recruited from the Allied Irish Bank Phase II (AIB Phase II) study that commenced in 1989 and consists of 387 bank employees (Table 1). Blood pressure was measured every 30 minutes for 24 hours using a validated monitor – the SpaceLabs 90207. The mean daytime and night time systolic (SBP) and diastolic (DBP) values were used for analysis in each case. Hypertension was defined as having daytime blood pressure of 135/85 mm Hg or greater. This group was collected between 1998 and 2002 and was between the ages of 30–70 years. It was noted if individuals consumed more than 2 alcohol-containing drinks per day.

The second were a group of 1649 patients with coronary disease (CHD), 1313 of whom were ascertained on the basis of having acute coronary syndromes (ACS) (myocardial infarction (MI) or unstable angina) and 336 as having stable angina (Table 2). MI was defined as chest pain of at least 20 minutes duration, along with previous or current electrocardiogram or serum enzyme changes diagnostic of MI. Unstable angina was defined as chest pain typical of angina occurring at rest or lasting at least 20 minutes and requiring hospitalization in a patient with known coronary artery disease based on a positive stress test or a coronary angiogram. Inclusion criteria for stable angina were chest pain occurring with exercise typical of angina in a patient with known coronary artery disease based on a coronary angiogram or a positive treadmill test. These patients were collected between the years 1999–2002 and were between the ages of 32 and 85 years. They were classified as having a history of hypertension requiring drug treatment or having no history of hypertension.

Informed consent and ethics approval from the Beaumont Hospital Ethics (Medical Research) Committee was obtained for all samples collected.

### *FMO3 *polymorphism genotyping

Three single nucleotide polymorphisms in the *FMO3 *gene were genotyped in the above populations. These were E158K (G472A), V257M (G769A) and E308G (A923G). The E158K polymorphism was genotyped using the primer GAAGGTGACCAAGTTCATGCTTGGCCTTACCTGGAAAGGACTT for the G allele and GAAGGTCGGAGTCAACGGATTTTTGGCCTTACCTGGAAAGGACT for the A allele. The V257M polymorphism was genotyped using the primer GAAGGTGACCAAGTTCATGCTCAGCCATCTCTGACTGGTTGTACA for the G allele and GAAGGTCGGAGTCAACGGATTAGCCATCTCTGACTGGTTGTACG for the A allele. The E308G polymorphism was genotyped using the primer GAAGGTGACCAAGTTCATGCTGCCTAACGTGAAGGAATTCACAGA for the A allele and GAAGGTCGGAGTCAACGGATTGCCTAACGTGAAGGAATTCACA for the G allele.

Genotyping was carried out using the Amplifluor™ method by K Biosciences . Genomic DNA was isolated from blood. Genotyping was performed in 384-well microplates using a fluorescence resonance energy transfer (FRET)-based genotyping method. Amplification was initiated using allele-specific primers and a common downstream primer. The allele-specific primers were tailed with unique sequences that create corresponding complementary sequences in the two amplicons. In the second round of amplification, quenched Universal Amplifluor™ primers (in a hairpin formation) were used. These primers contain 3' tails that specifically bind to the unique tailed sequences in the amplicons and continue amplification. In the final round of amplification, the action of the DNA polymerase opened up the hairpin structure and the quencher and reporter moieties are spatially separated. The excited reporter moiety emitted either red or green fluorescence, the colour of which depends on which nucleotide is at the polymorphism site. The fluorescence was quantified by a microplate reader and then analysed via an Excel macro to provide genotypes for each SNP. Confirmation of the validation of the mutation detection method was based on the use of known positive DNA controls (which had been sequenced) for the three polymorphisms provided by McGill University (Montreal).

### Haplotype analysis

Haplotypes for the 3 SNPs were inferred using a maximum likelihood approach and their association with the rank of the various mean blood pressure measurements in the AIB Phase II study group and their association with hypertension in the CVD group was determined using "haplo.score", a function written for the statistical package SPLUS 6.0[30]. This method used score tests for association between a quantitative trait and the haplotypes. The result of the association was given as a haplotype score. The haplotypes score was calculated using a general linear model, which permitted adjustment for age, sex and alcohol consumption.

### Statistical analysis

The 2 primary analyses were (1) the association between haplotypes of the 3 SNPs and mean daytime systolic blood pressure in the AIB Phase II study population and (2) the association between haplotypes and the hypertension status in the CVD patients.

The secondary analyses included (1) the association between genotypes and daytime systolic blood pressure in the AIB Phase II study, (2) the association between genotypes and hypertensive status in the CVD group, (3) the comparison of genotypic frequencies between the AIB Phase II study group, the hypertensives of the CVD group and the non-hypertensives of the CVD group, (4) the effect of haplotypes on mean daytime diastolic blood pressure, on mean night time systolic blood pressure and on mean night time diastolic blood pressure in the AIB Phase II study group, (5) the association between polymorphisms in the FMO3 gene and cardiovascular disease.

Haplo.score was used to look for any associations between haplotypes and daytime systolic blood pressure/hypertensive status. We only tested for relatively common haplotypes, and defined as those at an inferred frequency of greater than 5% in the populations. For all of the above analyses, statistical significance was determined when p < 0.05.

ANOVA was used to look for an association between the genotypes and mean daytime systolic blood pressure in the AIB Phase II study group. Both haplo.score and ANOVA were adjusted for age, sex and the number of alcohol units consumed per week.

Chi-squared analysis was used to look for the difference in genotype frequencies between the hypertensive and non-hypertensive patients in the CVD group. Linear regression was then used to adjust for age and sex. Chi-squared analysis was also used to compare genotypic frequencies between the AIB Phase II study group, the hypertensives from the CVD group and the non-hypertensives of the CVD group. Logistic regression analysis was used to predict if any of the SNPs influence the risk of cardiovascular disease, including diabetes, BMI, hypercholesterolemia, smoking status, age and gender in the model. All of the above analyses were repeated stratifying according to gender.

## Results

The AIB Phase II population consisted of 387 subjects, 224 (57.8%) of which were male. In comparison to the females, the males had on average higher blood pressure measurements, triglycerides and total cholesterol. Slightly more males in this group were smokers (Table 1).

The CVD group consisted of 1649 subjects, 691 (41.9%) of which were classified as having a history of hypertension requiring drug treatment. A greater percentage of those classified as hypertensive also had hypercholesterolemia and diabetes mellitus in comparison to those who were classified as non-hypertensive. As might be expected from a CVD group, most of the subjects were taking medication, with almost the same frequency of hypertensives and non-hypertensives taking each group of medications (Table 2). Blood pressure measurements are only presented to characterise, rather than compare, the groups, since medication is lowering blood pressure in most of the CHD group, but not in most of the AIB Phase II study group.

The most notable difference between the AIB Phase II study group and the CVD group is that there were a much greater proportion of current or ex smokers in the CVD group than in the AIB Phase II study group (73% vs. 30.1%).

The 3 SNPs were found to be in Hardy-Weinberg Equilibrium. 4 common haplotypes were inferred in the two populations (Table 3), with similar frequencies in the AIB Phase II study group, the hypertensives and in the non-hypertensives of the CVD group. However the haplotype KVG, which is the 2^nd ^most frequent haplotype in the AIB Phase II study group, was the 3^rd ^most frequent haplotype in the CVD group. However the difference in frequency was only 4%, which is most likely due to chance.

No statistically significant associations were found between any of the four common haplotypes and mean daytime systolic blood pressure in the AIB Phase II study (overall p = 0.65, 3 d.f) (See table 3). Nor was this statistically significant when adjusted for age, sex and daily alcohol intake (overall p = 0.729, 3 d.f) (See table 3). Neither did we find any statistically significant associations between any of the haplotypes and hypertension status in all subjects with and without adjustment of sex and age (see table 3). When stratified by gender, there were still no statistically significant associations between any of the specific haplotypes and daytime SBP in the AIB Phase II study (males overall p = 0.91, 3 df and females overall p = 0.61, 3 df) or with hypertension status in the CVD patients (males overall p = 0.59, 3 df and females overall p = 0.82, 3 df). Table 4 displays the genotypic frequencies of the 3 SNPs in the AIB Phase II population, in the hypertensives of the CVD group and in the non-hypertensives of the CVD group. When genotypic frequencies were compared among 3 groups, there were no statistically significant differences (E158K p = 0.30, V257M p = 0.47, E308G p = 0.15). There were no statistically significant associations between any of the individual genotypes with daytime SBP in the AIB Phase II study group, even when adjusted for age, sex and daily alcohol consumption (E158K p = 0.91, V257M p = 0.85, E308G p = 0.68).

The mean daytime SBP and mean daily alcohol consumption are broken down by genotype in table 5 and it is clear that there is not much difference between the genotype groups. Stratifying the analysis by gender, there was still no association between genotypic frequencies in the AIB Phase II study group (females E158K p = 0.92, V257M p = 0.86, E308G p = 0.13, males E158K p = 0.37, V257M p = 0.87, E308G p = 0.38).

There was no genotypic difference between hypertensive and non-hypertensive CVD subjects, even after adjustment for age and sex (E158K p = 0.84, V257M p = 0.44, E308G p = 0.91). When this analysis was stratified by gender, still no statistically significant differences are noted (females E158K p = 0.79, V257M p = 0.54, E308G p = 0.29, males E158K p = 0.71, V257M p = 0.22, E308G p = 0.66).

Testing for any associations between haplotype and daytime diastolic blood pressure, night time systolic blood pressure and night time diastolic blood pressure in the AIB Phase II study yielded no statistically significant associations (Table 6). Likewise it was shown by logistic regression that none of the SNPs were significant predictors for cardiovascular disease when the CVD and the AIB Phase II study groups were compared (E158K z = 0.60 p = 0.55, V257M z = -0.31 p = 0.76, E308G z = 0.49 p = 0.63), though diabetes was found to be a significant predictor.

## Discussion

The observation that a number of patients with TMAuria are hypertensive was the basis to the hypothesis that there may be an association between variants in the *FMO3 *gene and hypertension. The FMO3 enzyme has a broad substrate specificity, which includes catecholamines as a minor pathway[21,26]. Catecholamines are produced in response to stress and modulate heart rate and blood pressure. Variants of the *FMO3 *gene resulting in decreased enzymatic activity could result in decreased catabolism of catecholamines, which impact on blood pressure homeostasis. As catecholamines are at peak levels during the day, we elected to study the association between daytime SBP and variants in the *FMO3 *gene as a primary endpoint. In our analysis, we adjusted for alcohol use as tyramine is a substrate for FMO3 oxidation[26]. The FMO3 enzyme is also modulated by the sex hormones[28]. Females with moderate to severe TMAuria note exacerbations perimenstrually and it is proposed that decreases in FMO3 enzymatic activity resulting from polymorphisms could also be influenced by hormonal events[31]. For this reason the analysis was stratified by treating males and females as separate groups.

In this study, by constructing haplotypes based on the 3 SNPs, we discovered that none of the haplotypes were statistically significantly associated with either daytime systolic blood pressure in the AIB Phase II study group or with hypertensive status in the CVD group. There was also a lack of association between any of the genotypes of the SNPs with daytime SBP or with hypertension status. Neither did the stratified analyses show any statitically significant associations.

While the definition of hypertension in the CVD population provides a rather arbitrary estimation of hypertension, the 24-hour blood pressure measurements in the AIB Phase II study were carefully controlled. Even so, the results from both groups showed that the variants had no association with blood pressure or hypertension status.

Why are *FMO3 *variants maintained in population, and in particular the E158K variant? It is proposed that genes such as *FMO3*, a drug and toxin detoxicating gene, may have survived as balanced polymorphisms in an ancient human or primate population to neutralise the effect of harmful exposures[32,33]. Evolutionary changes in *FMO3 *may have evolved in different geographic locations to buffer changes in the highly polymorphic drug metabolising genes, which show substantial geographic and ethnic variation. It is possible that 'low penetrance' polymorphisms of *FMO3 *alone may not independently affect blood pressure homeostasis but severe loss of function causing mutations may unmask pressor effects of variation in other drug metabolising enzymes previously buffered by *FMO3*[34]. This hypothesis is supported by the observation that only some, but not all, TMAuria patients exhibit hypertensive symptoms. Therefore other common FMO genes may be potential hypertension candidates. There could be identified by typing other FMO genes in TMAuria patients with hypertensive symptoms or by directly assessing the candidate genes in a large study of hypertension.

## Conclusion

In conclusion, these data indicate that these three polymorphisms of the *FMO3 *gene inherited singly or in specific haplotype combinations do not represent an independent susceptibility risk for hypertension as an absolute trait or continuous variable in a representative Irish population.

## Authors' contributions

CD carried out the statistical analysis and drafted the manuscript. DCS assisted in the statistical analysis and participated in the study design. AS contributed to the interpretation of the hypertension data and participated in the study design. EOB designed the data collection for the AIB Phase II study and participated in the study design. DML helped draft the manuscript. JKOB led the genotypic analysis component. EPT conceived the study and helped to draft the manuscript. All authors read and approved the final manuscript.

## Pre-publication history

The pre-publication history for this paper can be accessed here:



## Figures and Tables

**Table 1:** Characteristics of AIB Phase II study group

	Male Mean/SD* (n = 224)	Female Mean/SD* (n = 163)
Age (years)	52.5 +- 9.2	47.4 +- 10.0
Weight (KG)	85.7 +- 16.1	66.3 +- 13.7
Height (cm)	177.5 +- 12.6	163.1 +- 11.3
Hypertensive	3.1%	1.2%
Daytime systolic BP (mmHg)	128.2 +- 10.3	118.3 +- 9.9
Daytime diastolic BP (mmHg)	80.8 +- 7.7	75.6 +- 7.3
Nighttime systolic BP (mmHg)	108.7 +- 9.8	101.5 +- 8.6
Nighttime diastolic BP (mmHg)	62.5 +- 7.5	58.1 +- 6.3
Total cholesterol (mmol/L)	5.7 +- 1.2	5.3 +- 1.2
Triglycerides (mmol/L)	1.5 +- 0.9	1.0 +- 0.6
Diabetes mellitus (%)	0.8%	0.6%
Smokers (Current/Ex) (%)	32.4%	27.9%
Medication:		
Antihypertensive (%)	3.6%	3.7%
Hormone replacement therapy	0%	21%
Anti-arrhythmic	0.5%	0%

*Standard deviation

**Table 2:** Characteristics of Cardiovascular disease group

	Hypertensive Mean/SD* (n = 691)	Non-hypertensive Mean/SD* (n = 958)
Age (years)	62.5 +/- 7.9	60.4 +/- 8.7
Weight (KG)	79.1 +/- 19.7	77.5 +/- 18.4
Height (cm)	160.3 +/- 34.9	163.1 +/- 34.3
Gender (males)	66.8%	83.6%
Hypercholesterolemia (%)	68.3%	51.7%
Diabetes mellitus (%)	12.9%	6.8%
Smokers (Current/Ex) (%)	69.9%	76.9%
Aspirin (%)	99.0%	99.2%
Statins (%)	96.3%	98.5%
B-Blockers (%)	95.9%	97.9%
Nitrates (%)	91.7%	95.4%
Calcium channel blockers (%)	92.2%	92.7%
ACE inhibitors (%)	92.2%	91.9%

*Standard deviation

**Table 3:** Associations between haplotypes in the AIB Phase II group with daytime systolic blood pressure and in the CVD group with hypertension status.

	AIB Phase II group					CVD group					
Haplotypes	Freq	Haplotype score^b^	p-value	Haplotype score^b*^	p-value*	Hypertensive freq	Non-hypertensive freq	Haplotype score^b^	p-value	Haplotype score^b†^	p-value^†^

EVE^a^	50.5%	0.497	0.62	0.392	0.70	53.5%	54.4%	-0.578	0.56	-0.715	0.47
KVG^a^	22.0%	0.638	0.52	0.629	0.51	18.0%	17.8%	0.206	0.84	0.433	0.66
KVE^a^	21.2%	-0.669	0.50	-0.687	0.49	21.5%	21.7%	-0.004	0.99	-0.073	0.94
EME^a^	6.3%	-0.954	0.34	-0.725	0.45	7.0%	6.1%	0.921	0.36	0.948	0.34
Overall p-value		0.65		0.73				0.80		0.79	

^a ^haplotypes defined for positions 158, 257 and 308 respectively

^b ^from the haplo.score method[30]

* Adjusted for age, sex and alcohol intake

^† ^Adjusted for age and sex

**Table 4:** Tabulation of genotype percentages and frequencies in groups

	Genotype (amino acid)	AIB Phase II study (387)	Hypertensive (775)	Non-hypertensive (984)
E158K	KK	19.4% (75)	14.6% (101)	16.0% (154)
	EK	45.7% (177)	47.7% (330)	45.6% (439)
	EE	33.8% (131)	35.1% (243)	37.1% (357)
	Missing	1.1% (4)	2.6% (18)	1.3% (12)
V257M	MM	1.0% (4)	0.4% (3)	0.3% (3)
	VM	11.1% (43)	12.3% (85)	11.3% (109)
	VV	86.3% (334)	86.4% (598)	87.6% (843)
	Missing	1.5% (6)	0.9% (6)	0.7% (7)
E308G	GG	5.7% (22)	3.3% (23)	3.3% (32)
	GE	31.0% (120)	28.2% (195)	28.5% (274)
	EE	61.2% (237)	66.9% (463)	66.8% (643)
	Missing	2.1% (8)	1.6% (11)	1.4% (13)

**Table 5:** Mean daytime systolic blood pressure and mean alcohol consumption broken down by genotype in the AIB Phase II study group.

	Genotype (amino acid)	Number subjects	Mean daytime sbp/SD*	Alcohol consumption +/- SD*
E158K	KK	75	126.71 +- 13.1	12.87 +- 13.8
	EK	177	125.87 +- 12.6	12.83 +- 12.4
	EE	131	126.61 +- 13.2	14.81 +- 14.1
V257M	MM	4	126.67 +- 9.3	14.00 +- 12.3
	VM	43	121.25 +- 14.7	15.50 +- 16.7
	VV	334	126.02 +- 12.7	13.24 +- 12.9
E308G	GG	22	129.32	15.65
	GE	120	125.82	11.87
	EE	237	126.18	14.26

* Standard deviation

**Table 6:** Summary of haplotype scores representing associations between blood pressure and haplotypes in the AIB phase II population showing overall statistical significance values

	Haplotypes (amino acid)	EVE^a^	KVG^a^	KVE^a^	EME^a^	Overall p-val
Daytime DBP	Haplotype score^b^	-0.136	-0.266	0.890	-0.726	0.76
	Haplotype score^b ^(adjusted*)	-0.361	-0.192	0.985	-0.565	0.77
Nighttime SBP	Haplotype score^b^	-0.657	1.676	-0.576	-0.586	0.40
	Haplotype score^b ^(adjusted*)	-0.803	1.676	-0.573	-0.294	0.42
Nighttime DBP	Haplotype score^b^	-1.313	1.313	0.665	-0.699	0.41
	Haplotype score^b ^(adjusted*)	-1.453	1.177	0.706	-0.260	0.47

* Adjusted for age, sex and alcohol intake

^a ^haplotypes defined from positions 128, 257 and 308 respectively

^b ^from the haplo.score method
